# Association of Atmospheric Particulate Matter and Ozone with Gestational Diabetes Mellitus

**DOI:** 10.1289/ehp.1408456

**Published:** 2015-03-20

**Authors:** Hui Hu, Sandie Ha, Barron H. Henderson, Tamara D. Warner, Jeffrey Roth, Haidong Kan, Xiaohui Xu

**Affiliations:** 1 Department of Epidemiology, College of Public Health and Health Professions and College of Medicine,; 2 Department of Environmental Engineering Sciences, Engineering School of Sustainable Infrastructure and Environment, and; 3 Department of Pediatrics, College of Medicine, University of Florida, Gainesville, Florida, USA; 4 Department of Environmental Health, School of Public Health, Fudan University, Shanghai, China

## Abstract

**Background:**

Ambient air pollution has been linked to the development of gestational diabetes mellitus (GDM). However, evidence of the association is very limited, and no study has estimated the effects of ozone.

**Objective:**

Our aim was to determine the association of prenatal exposures to particulate matter ≤ 2.5 μm (PM_2.5_) and ozone (O_3_) with GDM.

**Methods:**

We used Florida birth vital statistics records to investigate the association between the risk of GDM and two air pollutants (PM_2.5_ and O_3_) among 410,267 women who gave birth in Florida between 2004 and 2005. Individual air pollution exposure was assessed at the woman’s home address at time of delivery using the hierarchical Bayesian space–time statistical model. We further estimated associations between air pollution exposures during different trimesters and GDM.

**Results:**

After controlling for nine covariates, we observed increased odds of GDM with per 5-μg/m^3^ increase in PM_2.5_ (OR_Trimester1_ = 1.16; 95% CI: 1.11, 1.21; OR_Trimester2_ = 1.15; 95% CI: 1.10, 1.20; OR_Pregnancy_ = 1.20; 95% CI: 1.13, 1.26) and per 5-ppb increase in O_3_ (OR_Trimester1_ = 1.09; 95% CI: 1.07, 1.11; OR_Trimester2_ = 1.12; 95% CI: 1.10, 1.14; OR_Pregnancy_ = 1.18; 95% CI: 1.15, 1.21) during both the first trimester and second trimester as well as the full pregnancy in single-pollutant models. Compared with the single-pollutant model, the ORs for O_3_ were almost identical in the co-pollutant model. However, the ORs for PM_2.5_ during the first trimester and the full pregnancy were attenuated, and no association was observed for PM_2.5_ during the second trimester in the co-pollutant model (OR = 1.02; 95% CI: 0.98, 1.07).

**Conclusion:**

This population-based study suggests that exposure to air pollution during pregnancy is associated with increased risk of GDM in Florida, USA.

**Citation:**

Hu H, Ha S, Henderson BH, Warner TD, Roth J, Kan H, Xu X. 2015. Association of atmospheric particulate matter and ozone with gestational diabetes mellitus. Environ Health Perspect 123:853–859; http://dx.doi.org/10.1289/ehp.1408456

## Introduction

Gestational diabetes mellitus (GDM) is a common complication during pregnancy. It is defined as any degree of glucose intolerance with onset or first recognition during pregnancy (American Diabetes Association 2013). GDM complicates up to 14% of all pregnancies depending on the populations observed. More than 200,000 cases were reported annually in the United States (American Diabetes Association 2013). GDM has adverse effects on both the mother and the developing fetus. About one-third of women with GDM will eventually develop type 2 diabetes (Linné et al. 2002), and women with GDM also have higher long-term risks of cardiovascular diseases compared with those without GDM (Kitzmiller et al. 2007). In children, GDM has been associated with both perinatal and long-term adverse health outcomes such as macrosomia (Hughes et al. 1997), shoulder dystocia (Athukorala et al. 2007), birth injuries (Mitanchez 2010), sustained glucose tolerance impairment (Silverman et al. 1995), obesity (Pettitt et al. 1985), and impaired intellectual abilities (Rizzo et al. 1997). GDM has also been associated with metabolic disturbances in offspring of mothers with GDM (Boerschmann et al. 2010; Clausen et al. 2008; Lawlor et al. 2011), and the prevalence of type 2 diabetes or pre-diabetes at 18–27 years of age was almost eight times higher among offspring of women with GDM compared with other children in a case–control study (Clausen et al. 2008). Although previous studies have shown that treatment of GDM can reduce serious perinatal morbidity such as macrosomia at birth (Crowther et al. 2005), a recent study found no significant difference in body mass index (BMI) *z*-scores or BMI ≥ 85th percentile in children at 4–5 years of age whose mothers were treated for GDM (*n* = 94) compared with children whose mothers had GDM but received only routine care (*n* = 105) (Gillman et al. 2010). However, the sample size of this study was relatively small and may be underpowered.

Despite great improvements in air quality following the Clean Air Act (1963), air pollution remains a significant public health problem in the United States. According to the State of the Air 2013 report by the American Lung Association (2013), 41% of the population in the United States still lives in counties that have unhealthy levels of air pollution. Evidence on the effects of air pollution on diabetes mellitus in the general population has been reported in several recent epidemiological studies. A study of the Danish Diet, Cancer and Health cohort reported that traffic-related air pollution, using nitrogen dioxide (NO_2_) as a proxy, was associated with higher mortality from diabetes (Raaschou-Nielsen et al. 2013). Two studies in North America reported positive associations of NO_2_ and PM_2.5_ (particulate matter with diameter ≤ 2.5 μm) with the prevalence of diabetes (Brook et al. 2008; Pearson et al. 2010). In addition, positive associations have been found between air pollution and insulin resistance, the pathological hallmark underlying diabetes (Andersen et al. 2012; Chuang et al. 2011; Coogan et al. 2012; Kelishadi et al. 2009; Kim and Hong 2012; Krämer et al. 2010; Puett et al. 2011; Sun et al. 2013).

Although the biological mechanisms leading to GDM are still unclear, it is plausible that air pollution during pregnancy may increase the risk of GDM by inducing oxidative stress, and consequently inflammation, insulin resistance, dyslipidemia, and systemic metabolic dysfunction (Andersen et al. 2012; Chuang et al. 2011; Coogan et al. 2012; Everett et al. 2010; Hotamisligil et al. 1993; Kelishadi et al. 2009; Kim and Hong 2012; Krämer et al. 2010; Lamb and Goldstein 2008; Puett et al. 2011; Sun Y et al. 2006; Sun Z et al. 2013). Although evidence of adverse effects of air pollution on birth defects and pregnancy complications such as gestational hypertension has been widely reported in the last decade (Šrám et al. 2005; Xu et al. 2014), studies focusing on the association between ambient air pollution and GDM are still very limited. To our knowledge, only three previous studies have investigated air pollution and GDM. Malmqvist et al. (2013) reported a positive association between NO_x_ exposure and GDM, whereas an earlier study by van den Hooven et al. (2009) reported no association. A recent study found that exposure to PM_2.5_ and other traffic-related pollutants during pregnancy has been associated with impaired glucose tolerance but not GDM in women from Boston, Massachusetts, USA (Fleisch et al. 2014). Given the inconclusive results and limited types of pollutants examined in previous studies, investigation of the association between GDM and other criteria air pollutants such as ozone (O_3_) is warranted. In this study, we analyzed Florida birth vital statistics records for 410,267 women who gave birth during 2004–2005, to examine the association between the risk of GDM and two ambient air pollutants, PM_2.5_ and O_3_, assessed using the hierarchical Bayesian space–time statistical model (HBM) developed by the U.S. Environmental Protection Agency (EPA) and the Centers for Disease Control and Prevention’s (CDC) National Environmental Public Health Tracking Network (U.S. EPA 2014). We also investigated whether associations between exposure to air pollution and GDM varied among different gestational periods (trimesters and full pregnancy).

## Materials and Methods

*Study population*. We obtained birth record data from the Bureau of Vital Statistics and Office of Health Statistics and Assessment, Florida Department of Health (Jacksonville, FL; http://www.floridahealth.gov/certificates/certificates/). The data included all registered live births in Florida between 1 January 2004 and 31 December 2005 (*n* = 445,028). Births with maternal residential addresses outside Florida (*n* = 4,672) were excluded. We used ArcGIS V10.1 software (ESRI, Redlands, CA, USA) to geocode the mother’s residential address at birth, and 439,370 cases (99.8%) were successfully geocoded. Cases whose maternal residential address could not be geocoded were excluded (*n* = 986). We further excluded 937 cases because of missing values related to gestational age. In addition, we excluded women who had non-singleton deliveries (*n* = 13,367), previous preterm births (*n* = 5,591), or prepregnancy diabetes mellitus (*n* = 2,821). Births with congenital abnormalities (*n* = 5,450), with weight < 400 g (*n* = 240), or with a gestational age < 24 or > 42 weeks (*n* = 697) were also excluded. Following these exclusion criteria a total of 410,267 women remained in the study population. The research protocol for this study was approved by the Institutional Review Board at the University of Florida and the Florida Department of Health. The study was exempt from informed consent requirements because it involves no more than a minimal risk to the privacy of individuals and the research could not practicably be conducted without this exemption.

*Outcome assessment*. All pregnant women in Florida are requested to screen for GDM through an oral glucose challenge test (OGCT) between the 24th and 28th weeks of the pregnancy. This test requires each pregnant woman to drink about 5 oz of a syrupy glucose solution that contains 50 g of sugar and then have her blood drawn 1 hour after drinking the solution. If a blood glucose level reaches > 140 mg/dL 1 hr after the OGCT, it indicates the possibility of GDM. Then the pregnant woman is further referred to another 3-hr fasting 100-g oral glucose tolerance test (OGTT). The test measures fasting blood glucose level and blood glucose levels at 1, 2, and 3 hr after drinking the solution. The following values are considered to be abnormal during the OGTT: fasting blood glucose level ≥ 95 mg/dL, 1-hr blood glucose ≥ 180 mg/dL, 2-hr blood glucose ≥ 155 mg/dL, and 3-hr blood glucose ≥ 140 mg/dL. Pregnant women are classified as having GDM if two abnormal values are recorded during the OGTT (American Diabetes Association 2003).

*Air pollution exposure assessment*. Air pollution exposure data was obtained from the U.S. EPA and CDC’s National Environmental Public Health Tracking Network (2003–2005) (U.S. EPA 2014). The U.S. EPA provided the HBM data from 2001 to 2008 for two air pollutants, PM_2.5_ and O_3_, with spatial resolutions of 12 km × 12 km and 36 km × 36 km across the continental areas in the United States. Daily air pollution concentration for each grid was also included. Compared with the widely used air monitoring data from the U.S. EPA’s Air Quality System (AQS; http://www.epa.gov/airquality/airdata), the HBM data could provide pollutant values at unobserved locations across the entire spatial field of interest. The U.S. EPA has used two important advanced methods, the Community Multiscale Air Quality (CMAQ) model and the HBM (McMillan et al. 2010), to produce the interpolated concentrations of air pollutants in space and time. The HBM approach combines the AQS monitoring data with CMAQ modeled data, which include emission, meteorology, and chemical modeling components, to predict air quality data for a specific time and spatial scale (McMillan et al. 2010). Given the limited and sparsely located air monitors in Florida, we used the 12-km grid output from the HBM data, which can account for the poor spatial coverage of air monitoring data.

Each mother’s geocoded residential address at the time of her child’s birth was spatially linked to the corresponding grid of the HBM data. Exposures were calculated as daily concentrations averaged over each of the first two trimesters (trimester 1: 1–13 weeks; trimester 2: 14–26 weeks) and the full gestational period determined by gestational age and delivery date of each woman. Gestational age was determined mainly by ultrasound. When ultrasound data were not available, clinical examination or last menstrual period was used to estimate gestational age.

*Covariates*. Information on maternal characteristics such as age, race/ethnicity, marital status, pregnancy smoking status, season and year of conception, and prenatal care status was obtained directly from the births records. Maternal age at delivery was categorized into six groups, with 5-year increments for women 20–40 years old, as well as two additional groups for < 20 and ≥ 40 years old. Race/ethnicity was categorized as non-Hispanic white, non-Hispanic black, Mexican American, Puerto Rican, Cuban American, Haitian American, and others. In addition, a dichotomous variable was used to indicate marital status. Maternal education was divided into three categories: < high school, high school or equivalent, and > high school. Pregnancy smoking status was categorized into three levels based on self-reported number of cigarettes smoked per day during pregnancy: nonsmokers, smokers with < 10 cigarettes/day, and smokers with ≥ 10 cigarettes/day. Season [warm (June–November) or cool (December–May)] and year (2003, 2004, or 2005) of conception were also treated as categorical variables. Prenatal care status was categorized into five groups: no care, began in first trimester, second trimester, or third trimester, as well as an additional group for subjects with missing values. Furthermore, we extracted census block group–level median household income from the 2000 Census (http://www2.census.gov/census_2000/datasets), and linked it to each woman. Household income was categorized into quartiles (< US$29,663, US$29,663–US$38,056, US$38,056–US$49,375, and ≥ US$49,375). We also obtained cartographic boundary file for urban areas from the 2000 Census to determine the urbanization status (urban or rural) where each woman lived. No information was available on other risk factors for GDM such as maternal prepregnancy BMI, family history of type 2 diabetes, and low physical activity.

*Statistical analysis*. We examined the distribution of categorical covariates and continuous exposures between women with GDM and those without GDM. Logistic regression models were used to investigate the association between exposure to air pollution during different trimesters of pregnancy and risks of GDM. Subjects with missing values of maternal age (*n* = 45), race/ethnicity (*n* = 6), education (*n* = 3,821), or marital status (*n* = 83) were excluded, leaving 13,943 women with GDM out of a total of 406,334 women with complete covariate data. PM_2.5_ and O_3_ were analyzed as continuous variables. Both an unadjusted model and an adjusted model controlling for maternal age, race/ethnicity, education, marital status, prenatal care, season and year of conception, urbanization, and median household income at census block group level were used. Odds ratios (ORs) and 95% confidence intervals (CIs) (per 5-μg/m^3^ increase in PM_2.5_ or per 5-ppb increase in O_3_) were reported for each pollutant during specific pregnancy periods. Co-pollutant logistic models were also implemented to evaluate potential confounding by co-pollutants.

Sensitivity analyses. We conducted several sensitivity analyses to test the robustness of our results. First, to account for the potential bias created by using an indicator for missing data of prenatal care, we conducted multiple imputation for all missing data using chained equations (White et al. 2011). All covariates as well as exposure and outcome variables were included in the imputation process, and 50 imputed data sets were generated. Second, to account for the potential underdiagnoses of GDM, we assumed an underreported rate of 0.5% and 1.0% among women without GDM, and simulated data sets were generated by randomly assigning 0.5% and 1.0% of subjects without GDM as GDM cases with 500 repeats using the Monte Carlo method. Then we made the comparisons between the results from the simulated data and our original results to check whether the underdiagnosed cases have influenced the observed effects. Third, to account for the potential misclassification of exposure, we performed two sets of sensitivity analyses. In the first set of capture-area analyses, only women living within 5 mi from any AQS monitors were included, and two separated analyses were conducted for all eligible women and only for eligible women with nonmissing data for at least 75% of days. In the second set of analyses, we used interpolated 1-km × 1-km data for the exposure assessment. To create the 1-km × 1-km exposure field, we applied a bicubic spline to the 12-km × 12-km gridded HBM product and output on a 1-km × 1-km grid that included the original 12-km vertices. This approach provides finer resolution, but cannot reproduce sub–12-km concentration peaks or troughs. Fourth, we performed the analyses without adjusting for season of conception to account for the possibility that conception season may adjust away all seasonal influences on the variation in the pollutants such that only spatial differences were left, which might be much more easily confounded by socioeconomic status (SES)–related factors. We also performed the analyses after additionally adjusting for smoking during pregnancy. Finally, to account for the potential overadjusting of urbanization due to its correlation with air pollutants, we performed a stratified analyses by urban–rural areas. All statistical analyses were conducted using SAS V9.3 (SAS Institute Inc., Cary, NC, USA).

## Results

Of the 410,267 women included in this study, 14,032 (3.4%) had GDM, including 406,334 with complete data for all covariates (*n* = 13,943 with GDM). Table 1 shows the distribution of exposures to PM_2.5_ and O_3_ for each pregnancy period analyzed in this study. Women with GDM had slightly higher levels of PM_2.5_ and O_3_ exposure compared with those without GDM during all pregnancy periods (all *p* < 0.001). Weak correlations were observed between PM_2.5_ and O_3_ in all gestational periods (Pearson’s correlation coefficients range from 0.21 to 0.39).

**Table 1:** Exposure information concerning PM_2.5_ and O_3_ by GDM status among women who gave birth in 2004–2005 in Florida, USA (*n *= 14,032 with GDM, *n *= 396,235 without GDM, and total *n *= 410,267).

Exposure/statistics	Trimester 1			Trimester 2			Full pregnancy		
	GDM	No GDM	Total	GDM	No GDM	Total	GDM	No GDM	Total
PM_2.5_ (μg/m^3^)
Mean ± SD	9.84 ± 2.16	9.72 ± 2.07	9.73 ± 2.07	9.94 ± 2.09	9.88 ± 2.06	9.88 ± 2.06	10.03 ± 1.71	9.93 ± 1.67	9.93 ± 1.67
Median	9.75	9.64	9.65	9.87	9.76	9.76	9.97	9.90	9.91
IQR	2.68	2.61	2.61	2.63	2.61	2.61	2.06	2.02	2.02
O_3_ (ppb)
Mean ± SD	37.71 ± 6.14	37.20 ± 6.04	37.22 ± 6.04	38.17 ± 6.10	37.52 ± 6.10	37.54 ± 6.10	37.85 ± 4.01	37.38 ± 4.10	37.40 ± 4.10
Median	36.73	36.48	36.48	37.65	36.92	36.95	38.40	37.82	37.84
IQR	8.24	7.82	7.83	8.46	7.99	8.00	6.94	7.10	7.09
Correlation between PM_2.5_ and O_3_	0.39	0.39	0.39	0.35	0.34	0.34	0.21	0.22	0.22
IQR, interquartile range.									

Table 2 shows the demographic characteristics of women by GDM status. Women with GDM were older and less likely to belong to non-Hispanic black racial/ethnic categories. Higher proportions of women with GDM were married and had higher education and income levels. GDM cases were more likely among women who started prenatal care early and whose conception began in the warm season or recent years.

**Table 2:** Maternal characteristics by GDM status among women who gave birth in 2004–2005 in Florida, USA [*n* (%)].

Maternal characteristic	GDM (*n *= 14,032)	No GDM (*n *= 396,235)	Total(*n *= 410,267)
Maternal age (years)
< 20	451 (3.2)	44,064 (11.1)	44,515 (10.9)
20–24	2,125 (15.1)	103,600 (26.2)	105,725 (25.8)
25–29	3,466 (24.7)	103,679 (26.2)	107,145 (26.1)
30–34	4,265 (30.4)	87,758 (22.2)	92,023 (22.4)
35–39	2,844 (20.3)	44,608 (11.3)	47,452 (11.6)
≥ 40	880 (6.3)	12,482 (3.2)	13,362 (3.3)
Missing	1 (0.0)	44 (0.0)	45 (0.0)
Race/ethnicity
Non-Hispanic white	6,674 (47.6)	188,029 (47.5)	194,703 (47.5)
Non-Hispanic black	2,041 (14.6)	70,355 (17.8)	72,396 (17.7)
Mexican American	1,253 (8.9)	28,370 (7.2)	29,623 (7.2)
Puerto Rican	634 (4.5)	18,831 (4.8)	19,465 (4.7)
Cuban American	590 (4.2)	20,123 (5.1)	20,713 (5.1)
Haitian American	541 (3.9)	12,573 (3.2)	13,114 (3.2)
Other	2,299 (16.4)	57,948 (14.6)	60,247 (14.7)
Missing	0 (0.0)	6 (0.0)	6 (0.0)
Maternal education
< High school	2,524 (18.0)	83,066 (21.0)	85,590 (20.9)
High school or equivalent	4,207 (30.0)	126,013 (31.8)	130,220 (31.7)
> High school	7,213 (51.4)	183,423 (46.3)	190,636 (46.5)
Missing	88 (0.6)	3,733 (0.9)	3,821 (0.9)
Marital status
Married	9,697 (69.1)	232,727 (58.7)	242,424 (59.1)
Not married	4,335 (30.9)	163,425 (41.2)	167,760 (40.9)
Missing	0 (0.0)	83 (0.0)	83 (0.0)
Smoking during pregnancy
No	12,769 (91.0)	360,016 (90.9)	372,785 (90.9)
Yes, < 10 cigarettes/day	483 (3.4)	14,163 (3.6)	14,646 (3.6)
Yes, ≥ 10 cigarettes/day	581 (4.1)	16,852 (4.3)	17,433 (4.3)
Missing	199 (1.4)	5,204 (1.3)	5,403 (1.3)
Season of conception
Warm	6,942 (49.5)	192,430 (48.6)	199,372 (48.6)
Cool	7,090 (50.5)	203,805 (51.4)	210,895 (51.4)
Year of conception
2003	4,131 (29.4)	142,945 (36.1)	147,076 (35.9)
2004	7,479 (53.3)	199,682 (50.4)	207,161 (50.5)
2005	2,422 (17.3)	53,608 (13.5)	56,030 (13.7)
Prenatal care began
No care	59 (0.4)	4,987 (1.3)	5,046 (1.2)
First trimester	7,698 (54.9)	188,869 (47.7)	196,567 (47.9)
Second trimester	2,022 (14.4)	57,504 (14.5)	59,526 (14.5)
Third trimester	570 (4.1)	14,115 (3.6)	14,685 (3.6)
Missing	3,683 (26.3)	130,760 (33.0)	134,443 (32.8)
Residential area
Urban	12,017 (85.6)	342,936 (86.6)	354,953 (86.5)
Rural	2,015 (14.4)	53,299 (13.5)	55,314 (13.5)
Median household income (US$)
< 29,663	3,326 (23.7)	99,224 (25.0)	102,550 (25.0)
29,663–38,056	3,494 (24.9)	99,047 (25.0)	102,541 (25.0)
38,056–49,375	3,648 (26.0)	98,825 (24.9)	102,473 (25.0)
≥ 49,375	3,564 (25.4)	99,139 (25.0)	102,703 (25.0)

Table 3 provides the unadjusted and adjusted ORs of single-pollutant logistic regression models predicting GDM from exposure to PM_2.5_ and O_3_ during different pregnancy periods. After controlling for all nine covariates, increased odds of GDM for a 5-μg/m^3^ increase in PM_2.5_ were observed during both the first and second trimesters (OR_Trimester1_ = 1.16; 95% CI: 1.11, 1.21; OR_Trimester2_ = 1.15; 95% CI: 1.10, 1.20); and the full pregnancy (OR = 1.20; 95% CI: 1.13, 1.26). Associations were also found between GDM and O_3_. The odds of GDM were higher for a 5-ppb increase in exposure to O_3_ during the first and second trimesters (OR_Trimester1_ = 1.09; 95% CI: 1.07, 1.11; OR_Trimester2_ = 1.12; 95% CI: 1.10, 1.14), and over the course of the entire pregnancy (OR = 1.18; 95% CI: 1.15, 1.21).

**Table 3:** ORs (95% CIs) for risk of GDM by air pollutants (PM_2.5_ and O_3_) and pregnancy period of exposure among women who gave birth in 2004–2005 in Florida, USA.

Exposure	*n* (GDM/total)	Unadjusted OR (95% CI)	*n* (GDM/total)^*a*^	Adjusted OR^*b*^ (95% CI)
PM_2.5_ (per 5 μg/m^3^)
Trimester 1	14,032/410,267	1.15 (1.10, 1.19)	13,943/406,334	1.16 (1.11, 1.21)
Trimester 2	14,032/410,267	1.08 (1.04, 1.12)	13,943/406,334	1.15 (1.10, 1.20)
Full pregnancy	14,032/410,267	1.19 (1.13, 1.25)	13,943/406,334	1.20 (1.13, 1.26)
O_3_ (per 5 ppb)
Trimester 1	14,032/410,267	1.07 (1.06, 1.09)	13,943/406,334	1.09 (1.07, 1.11)
Trimester 2	14,032/410,267	1.09 (1.08, 1.10)	13,943/406,334	1.12 (1.10, 1.14)
Full pregnancy	14,032/410,267	1.16 (1.13, 1.18)	13,943/406,334	1.18 (1.15, 1.21)
^***a***^Women with complete data for all covariates. ^***b***^Adjusted for maternal age, race, education, marital status, season of conception, year of conception, prenatal care began, urbanization, and median household income.				

The results from the sensitivity analyses are presented in the Supplemental Material. Specifically, multiple imputation was conducted in the first set of sensitivity analyses to assess the potential effects of missing data on the results, and we observed ORs almost identical to the original results (see Supplemental Material, Table S1). Second, the Monte Carlo method was used to generate two sets of simulated data sets assuming the underreported rate of GDM was 0.5% and 1.0%. Compared with the original results, the ORs from the simulated data sets slightly attenuated, but the conclusions remain consistent (see Supplemental Material, Table S2). Third, we examined the effects of potential misclassifications of exposure on the results separately using capture-area analyses and the interpolated 1-km × 1-km HBM data. Compared with the original results, we observed comparable ORs for O_3_ during the second trimester and PM_2.5_ during the second trimester and full pregnancy period in the capture-area analyses. However, attenuated ORs were observed for O_3_ during the first trimester and the full pregnancy period, and no significant association was found for PM_2.5_ in the first trimester. On the other hand, the results from the interpolated HBM in the 1-km × 1-km resolution showed consistent ORs with the original results (see Supplemental Material, Table S3). Fourth, we assessed whether adjusting for smoking during pregnancy may bias the findings, and we observed consistent ORs with the original results. We also analyzed the data without adjusting for season of conception, and consistent results were observed except for the slightly attenuated OR for O_3_ in the first trimester (see Supplemental Material, Table S4). Last, a stratified analyses by urbanization was performed to examine the potential overadjustment of it, and no statistically significant difference was observed between the nonstratified results and the stratified results (see Supplemental Material, Table S5).

**Figure 1:**
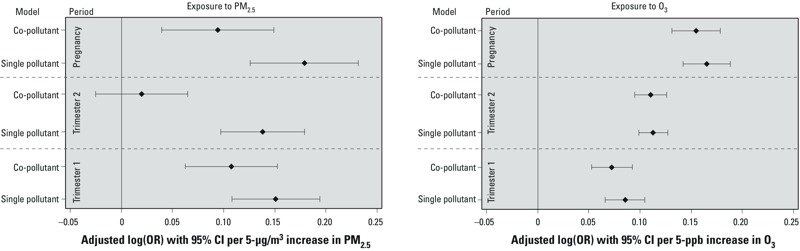
Adjusted log(OR) for risk of GDM with per 5 units increase in gestational exposure to pollutant for single- and co-pollutant models among women who gave birth in 2004–2005 in Florida, USA. Diamonds reflect the central estimate; whiskers represent the 95% CIs.

The results of the co-pollutant models are provided in Supplemental Material, Table S6. Figure 1 compares the results obtained from single- and co-pollutant continuous models. The ORs for O_3_ after adjusting for PM_2.5_ were almost identical to the ORs from the single-pollutant model. However, the ORs for PM_2.5_ during the first trimester and the full pregnancy attenuated after adjusting for O_3_, and no association was observed for PM_2.5_ during the second trimester in the co-pollutant model (OR = 1.02; 95% CI: 0.98, 1.07 compared with OR = 1.15; 95% CI: 1.10, 1.20 from the single-pollutant model).

## Discussion

We examined the association of GDM with PM_2.5_ and O_3_ during different pregnancy periods using Florida birth vital statistics records and the U.S. EPA and CDC’s HBM air pollution data, which have both good spatial and temporal coverage. When assessed in single-pollutant models, GDM was significantly associated with per 5-unit increases in both PM_2.5_ and O_3_ during the first and second trimesters and the full pregnancy. The associations were also found in co-pollutant models for PM_2.5_ exposure during the first trimester and O_3_ exposure during all pregnancy periods we examined. The associations persisted with adjustment for confounding by maternal characteristics such as age, race/ethnicity, education, marital status, prenatal care, season and year of conception, urbanization, and median household income at census block group level. The results of this study add to the emerging evidence linking air pollution exposure during pregnancy to pregnancy complications such as GDM.

The causal mechanisms underlying the associations between air pollution and GDM are still unclear; however, the results observed in this study are consistent with several potential pathways suggested by previous studies. Ambient air pollutants such as PM and O_3_ have been reported to be associated with increased insulin resistance, dyslipidemia, and systemic metabolic dysfunction (Andersen et al. 2012; Chuang et al. 2011; Coogan et al. 2012; Kelishadi et al. 2009; Kim and Hong 2012; Krämer et al. 2010; Puett et al. 2011; Sun et al. 2013), which are all precursors associated with GDM. PM contains many toxic chemicals that are regarded as reactive oxygen species (ROS) (Lemaire and Livingstone 1997; Sun et al. 2006), which can cause oxidative damage on target tissues (Ames et al. 1993). The imbalance between the production of ROS and antioxidant defenses is acknowledged as one of the main causes of insulin signaling–pathways alterations (Lamb and Goldstein 2008), and a number of studies have linked ROS to insulin resistance (Goldstein et al. 2005; Schulz et al. 2007). In addition, a recent animal study also showed O_3_’s ability to induce glucose intolerance and systemic metabolic effects (Bass et al. 2013). In their study on young and aged Brown Norway rats, Bass et al. (2013) observed increased α_2_-macroglobulin, adiponectin, and osteopontin as well as decreased phosphorylated insulin receptor substrate-1 in liver and adipose tissues following acute O_3_ exposure. Endoplasmic reticular stress was suggested to be the consequence of O_3_-induced acute metabolic impairment. Furthermore, another potential pathway induced by air pollution is inflammation, which may also lead to the development of insulin resistance (Everett et al. 2010; Hotamisligil et al. 1993).

Cigarette smoking has been widely reported to be associated with type 2 diabetes (Willi et al. 2007; Zhu et al. 2014), and we initially considered it as a potential confounder in our analyses. However, given the fact that smoking is not generally considered a risk factor for GDM as well as the consistent results we observed with or without adjusting for it in the sensitivity analyses, we finally present results without adjusting for smoking. In addition, although the underlying mechanisms remain unknown, our findings that air pollution may have an impact on risk of GDM does not conflict with the null association between smoking and GDM because their toxic components are largely different.

Our study has several strengths. First, compared with the air monitoring data that have been widely used in other studies, the daily temporal resolution and the 12-km × 12-km spatial resolution of HBM air pollution data used in this study allowed us to estimate mean air pollution concentrations during different pregnancy periods without excluding subjects not covered by air monitors, thus reducing the potential for selection bias. Second, previous studies focused only on small areas and examined limited types of air pollutants. With the HBM air pollution data, we were able to include all pregnant women in the study period throughout the entire state of Florida and investigate the association between GDM and two common air pollutants, PM_2.5_ and O_3_, which have not been reported in the extant literature. Furthermore, we used both single- and co-pollutant models to examine the association between air pollution and GDM. The robust results of O_3_ observed from different models suggest that it may have effects on GDM independent of PM_2.5_. This finding is consistent with recent experimental studies (Bass et al. 2013). It is also consistent with the positive association found between NO_x_ (nitrogen oxides) and GDM (Malmqvist et al. 2013) because NO_x_ is one main precursor of O_3_ (Sillman 1999). Finally, the robust results from the sensitivity analyses suggested that the study was not likely to be largely biased by the missing data, exposure and outcome misclassifications, and underadjustment of smoking during pregnancy or overadjustments of season of conception and urbanization.

This study had several limitations. First, it is possible that GDM may be underdiagnosed in the source vital statistics records. Second, as reported by the American Diabetes Association (2013), more women of childbearing age have type 2 diabetes due to an epidemic of obesity and diabetes in recent years. This trend may result in an increase in the number of women with undiagnosed type 2 diabetes, leading to potential misclassification of GDM in this study. However, because our study period covered the years 2003–2005, our results are less likely to be biased by the effects of undiagnosed diabetes in recent years. Third, information on daily mobility and behavior patterns was not available for this study. The absence of these factors may introduce misclassifications of exposure. A high correlation between personal monitored air pollution measurement and monthly aggregated modeled air pollution measurement has been reported in a cohort of 85 pregnant women in Manchester and Blackpool, United Kingdom (Hannam et al. 2013), although we cannot assess its comparability to our study due to the lack of daily mobility data. Fourth, residential mobility during pregnancy was also not available in this study. It may be possible that some subjects in this study lived elsewhere in the early stage of their pregnancy and thus were exposed to different levels of air pollution. Fifth, although the use of HBM air pollution data can avoid selection bias, the 12-km × 12-km resolution is very crude. Although the spatial variability of O_3_ is low, the variability of PM_2.5_ may be a concern, which includes a large-scale regional component and a local source component. Isakov et al. (2012) suggested that the regional component provides most of the mass, going as far as to use PM_2.5_ as an example of spatially homogeneous pollutants. Therefore, exposure to PM_2.5_ is not likely to have extremely fine-scale variability in most places in Florida. In addition, highly variable exposure fields would also be inappropriate for use with residential address only. However, future studies with higher spatial resolution modelling data and detailed time–activity patterns are warranted. Sixth, although several important confounders have been included in this study, no information on such other risk factors for GDM as prepregnancy BMI, family history of type 2 diabetes, and physical activity was available. These unadjusted factors may influence the results. For example, if obese women are more likely to live in areas with higher air pollution, the observed effects of air pollution on GDM in this study may be overestimated without controlling for this factor. In addition, low population densities, poor street connectivity, and lack of sidewalks in rural areas have been linked to increased physical inactivity and obesity (Eberhardt and Pamuk 2004), which are also characterized by having higher O_3_ concentrations. Although we adjusted for urbanization in this study, residual confounding may still exist. Thus, future studies with more detailed information on these factors are warranted to confirm our findings. Another potential limitation of the study is the unavailability of traffic noise data. Traffic noise induces a stress response and disturbs sleep, which has been associated with higher levels of stress hormone and decreased insulin levels and sensitivity (Sørensen et al. 2013). Both maternal stress and/or disturbances of sleep during pregnancy increase the risk of GDM. Because road traffic is the main source for both air pollution with PM_2.5_ and noise in urban areas, the mutual confounding is a concern. Finally, the results observed in birth registry data may also be influenced by the fixed cohort bias (Strand et al. 2011). Fixed cohort bias is a type of selection bias that could happen in retrospective cohorts with a fixed start and end date when short pregnancies are missed at the start of the study, and longer pregnancies are missed at the end. Because GDM is linked to preterm birth, fixed cohort bias may exist if GDM cases are more likely to be excluded at the beginning and to be included at the end of the study. However, given the facts that fixed cohort bias tends to decrease when the study has longer study period and/or when it has a day and month of the start date (i.e., 1 January 2004) just before day and month of the end date (i.e., 31 December 2005), the potential for this bias was reduced in this study.

## Conclusion

Using Florida birth vital statistics records, we observed a positive association between increased prevalence of GDM and exposure to PM_2.5_ and O_3_ during each trimester of pregnancy and the full pregnancy among women giving birth in 2004 and 2005. This study suggests the need for greater attention on stronger air pollution controls to improve the health of pregnant women and their offspring.

## Supplemental Material

(327 KB) PDF
