# Supplementary material for: Association of Atmospheric Particulate Matter and Ozone with Gestational Diabetes Mellitus
Source: Environ Health Perspect. 2015 Mar 20;123(9):853–9. doi: 10.1289/ehp.1408456 (PMC4559952; doi:10.1289/ehp.1408456)
Supplement: (327 KB) PDF [file ehp.1408456.s001.acco.pdf]

Environ Health Perspect
doi: 10.1289/ehp.1408456

Note to Readers: ***EHP*** **strives to ensure that all journal content is accessible to all readers. However, some figures and Supplemental Material published in** ***EHP*** **articles may not conform to 508 standards due to the complexity of the information being presented. If you need assistance accessing journal content, please contact ehp508@niehs.nih.gov. Our staff will work with you to assess and meet your accessibility needs within 3 working days.**

Supplemental Material

Association of Atmospheric Particulate Matter and Ozone with Gestational Diabetes Mellitus

Hui Hu, Sandie Ha, Barron H. Henderson, Tamara D. Warner, Jeffrey Roth, Haidong Kan, and Xiaohui Xu

Table of Contents

Table S1. Comparisons of ORs for risk of gestational diabetes mellitus (GDM) by air pollutants ($PM_{2.5}$ and O_3) and pregnancy period of exposure between original findings and results with multiple imputation among women who gave birth from 2004 to 2005 in Florida, USA.

Table S2. ORs for risk of gestational diabetes mellitus (GDM) by air pollutants ($PM_{2.5}$ and O_3) and pregnancy period of exposure by different underreported rate among women who gave birth from 2004 to 2005 in Florida, USA.

Table S3. ORs for risk of gestational diabetes mellitus (GDM) by air pollutants ($PM_{2.5}$ and O_3) and pregnancy period of exposure among women who gave birth from 2004 to 2005 in Florida, USA.

Table S4. Comparisons of ORs for risk of gestational diabetes mellitus (GDM) by air pollutants ($PM_{2.5}$ and O_3) and pregnancy period of exposure with and without adjusting for season of conception or smoking during pregnancy among women who gave birth from 2004 to 2005 in Florida, USA.

Table S5. Comparisons of ORs for risk of gestational diabetes mellitus by pregnancy period of exposure from non-stratified models and stratified models by urbanization among women who gave birth from 2004 to 2005 in Florida, USA.

Table S6. ORs for risk of gestational diabetes mellitus by pregnancy period of exposure from co-pollutant ($PM_{2.5}$ and O_3) models among women who gave birth from 2004 to 2005 in Florida, USA.

Table S1. Comparisons of ORs for risk of gestational diabetes mellitus (GDM) by air pollutants ($PM_{2.5}$ and O_3) and pregnancy period of exposure between original findings and results with multiple imputation among women who gave birth from 2004 to 2005 in Florida, USA.

	Without Multiple Imputation		With Multiple Imputation	
**Exposure**	**n (GDM/Total)[a]**	**Adjusted OR[b] (95% CI)**	**n (GDM/Total)**	**Adjusted OR[b] (95% CI)**
**$PM_{2.5}$ (per 5 μg/m^3)**				
Trimester 1	13,943/406,334	1.16(1.11, 1.21)	14,032/410,267	1.16(1.11, 1.21)
Trimester 2	13,943/406,334	1.15(1.10, 1.20)	14,032/410,267	1.14(1.09, 1.19)
Full pregnancy	13,943/406,334	1.20(1.13, 1.26)	14,032/410,267	1.19(1.12, 1.25)
**O_3 (per 5 ppb)**				
Trimester 1	13,943/406,334	1.09(1.07, 1.11)	14,032/410,267	1.09(1.07, 1.12)
Trimester 2	13,943/406,334	1.12(1.10, 1.14)	14,032/410,267	1.12(1.11, 1.14)
Full pregnancy	13,943/406,334	1.18(1.15, 1.21)	14,032/410,267	1.19(1.16, 1.22)

[a]Women with complete data for all covariates. [b]Adjusted for maternal age, race, education, marital status, season of conception, year of conception, prenatal care began, urbanization, and median household income.

Table S2. ORs for risk of gestational diabetes mellitus (GDM) by air pollutants ($PM_{2.5}$ and O_3) and pregnancy period of exposure by different underreported rate among women who gave birth from 2004 to 2005 in Florida, USA.

	**Original Results**	**Results from Simulation (500 repeats)**	
		**Underreported Rate: 0.5%**	**Underreported Rate: 1.0%**
**Exposure**	**Adjusted OR[a] (95% CI)**	**Adjusted OR[a] (95% CI)**	**Adjusted OR[a] (95% CI)**
**$PM_{2.5}$ (per 5 μg/m^3)**			
Trimester 1	1.16(1.11, 1.21)	1.14(1.10, 1.19)	1.12(1.08, 1.17)
Trimester 2	1.15(1.10, 1.20)	1.13(1.09, 1.18)	1.12(1.07, 1.16)
Full pregnancy	1.20(1.13, 1.26)	1.17(1.11, 1.23)	1.15(1.09, 1.20)
**O_3 (per 5 ppb)**			
Trimester 1	1.09(1.07, 1.11)	1.08(1.06, 1.10)	1.07(1.05, 1.08)
Trimester 2	1.12(1.10, 1.14)	1.10(1.09, 1.12)	1.09(1.08, 1.11)
Full pregnancy	1.18(1.15, 1.21)	1.15(1.13, 1.18)	1.13(1.11, 1.16)

[a]Adjusted for maternal age, race, education, marital status, season of conception, year of conception, prenatal care began, urbanization, and median household income.

Table S3. ORs for risk of gestational diabetes mellitus (GDM) by air pollutants ($PM_{2.5}$ and O_3) and pregnancy period of exposure among women who gave birth from 2004 to 2005 in Florida, USA.

Exposure	Exposure assessed by AQS monitors: All subjects within 5 miles of monitor		Exposure assessed by AQS monitors: Subjects within 5 miles of monitor and with nonmissing data for at least 75% of days		Exposure assessed by 1km×1km Interpolated HBM		Exposure assessed by 12km×12km HBM	
	n (GDM/Total)[a]	Adjusted OR[b] (95% CI)	n (GDM/Total)[a]	Adjusted OR[b] (95% CI)	n (GDM/Total)[a]	Adjusted OR[b] (95% CI)	n (GDM/Total)[a]	Adjusted OR[b] (95% CI)
**$PM_{2.5}$ (per 5 μg/m^3)**								
Trimester 1	3,879/117,824	0.98(0.89, 1.07)	2,197/67,152	1.00(0.88, 1.13)	13,943/406,334	1.17(1.12, 1.22)	13,943/406,334	1.16(1.11, 1.21)
Trimester 2	3,956/120,889	1.21(1.12, 1.31)	2,254/68,335	1.40(1.26, 1.57)	13,943/406,334	1.15(1.10, 1.19)	13,943/406,334	1.15(1.10, 1.20)
Full pregnancy	4,054/124,900	1.14(1.04, 1.26)	2,224/68,208	1.33(1.17, 1.51)	13,943/406,334	1.20(1.13, 1.27)	13,943/406,334	1.20(1.13, 1.26)
**O_3 (per 5 ppb)**								
Trimester 1	3,482/107,588	1.02(0.99, 1.05)	3,258/101,280	1.03(1.00, 1.07)	13,943/406,334	1.09(1.07, 1.11)	13,943/406,334	1.09(1.07, 1.11)
Trimester 2	3,513/108,481	1.07(1.04, 1.09)	3,277/102,020	1.08(1.05, 1.11)	13,943/406,334	1.12(1.11, 1.14)	13,943/406,334	1.12(1.10, 1.14)
Full pregnancy	3,589/110,040	1.04(1.00, 1.08)	3,340/103,380	1.09(1.05, 1.14)	13,943/406,334	1.18(1.16, 1.21)	13,943/406,334	1.18(1.15, 1.21)

[a]Women with complete data for all covariates. [b]Adjusted for maternal age, race, education, marital status, season of conception, year of conception, prenatal care began, urbanization, and median household income.

Table S4. Comparisons of ORs for risk of gestational diabetes mellitus (GDM) by air pollutants ($PM_{2.5}$ and O_3) and pregnancy period of exposure with and without adjusting for season of conception or smoking during pregnancy among women who gave birth from 2004 to 2005 in Florida, USA.

	Original Results		Without Adjustment of Season of Conception		With Adjustment of Smoking during Pregnancy	
**Exposure**	**n (GDM/Total)[a]**	**Adjusted OR[b] (95% CI)**	**n (GDM/Total)[a]**	**Adjusted OR[c] (95% CI)**	**n (GDM/Total)[a]**	**Adjusted OR[d] (95% CI)**
**$PM_{2.5}$ (per 5 μg/m^3)**						
Trimester 1	13,943/406,334	1.16(1.11, 1.21)	13,943/406,334	1.11(1.07, 1.16)	13,750/401,181	1.17(1.12, 1.22)
Trimester 2	13,943/406,334	1.15(1.10, 1.20)	13,943/406,334	1.16(1.11, 1.20)	13,750/401,181	1.16(1.11, 1.21)
Full pregnancy	13,943/406,334	1.20(1.13, 1.26)	13,943/406,334	1.20(1.14, 1.26)	13,750/401,181	1.21(1.14, 1.27)
**O_3 (per 5 ppb)**						
Trimester 1	13,943/406,334	1.09(1.07, 1.11)	13,943/406,334	1.03(1.01, 1.05)	13,750/401,181	1.09(1.07, 1.11)
Trimester 2	13,943/406,334	1.12(1.10, 1.14)	13,943/406,334	1.12(1.10, 1.13)	13,750/401,181	1.12(1.11, 1.14)
Full pregnancy	13,943/406,334	1.18(1.15, 1.21)	13,943/406,334	1.18(1.15, 1.20)	13,750/401,181	1.18(1.15, 1.21)

[a]Women with complete data for all covariates. [b]Adjusted for maternal age, race, education, marital status, season of conception, year of conception, prenatal care began, urbanization, and median household income. [c]Adjusted for maternal age, race, education, marital status, year of conception, prenatal care began, urbanization, and median household income. [d]Adjusted for maternal age, race, education, marital status, smoking during pregnancy, season of conception, year of conception, prenatal care began, urbanization, and median household income.

Table S5. Comparisons of ORs for risk of gestational diabetes mellitus by pregnancy period of exposure from non-stratified models and stratified models by urbanization among women who gave birth from 2004 to 2005 in Florida, USA.

	Stratified Results				Original Results (Non-stratified)	
	Urban Areas		Rural Areas			
**Exposure**	**n (GDM/Total)[a]**	**Adjusted OR[b] (95% CI)**	**n (GDM/Total)[a]**	**Adjusted OR[b] (95% CI)**	**n (GDM/Total)[a]**	**Adjusted OR[c] (95% CI)**
**$PM_{2.5}$ (per 5 μg/m^3)**						
Trimester 1	11,936/351,222	1.15(1.10, 1.21)	2,007/55,112	1.22(1.09, 1.36)	13,943/406,334	1.16(1.11, 1.21)
Trimester 2	11,936/351,222	1.14(1.09, 1.19)	2,007/55,112	1.22(1.09, 1.36)	13,943/406,334	1.15(1.10, 1.20)
Full pregnancy	11,936/351,222	1.18(1.12, 1.25)	2,007/55,112	1.32(1.14, 1.52)	13,943/406,334	1.20(1.13, 1.26)
**O_3 (per 5 ppb)**						
Trimester 1	11,936/351,222	1.09(1.07, 1.11)	2,007/55,112	1.08(1.02, 1.15)	13,943/406,334	1.09(1.07, 1.11)
Trimester 2	11,936/351,222	1.12(1.11, 1.14)	2,007/55,112	1.10(1.05, 1.14)	13,943/406,334	1.12(1.10, 1.14)
Full pregnancy	11,936/351,222	1.17(1.15, 1.20)	2,007/55,112	1.24(1.14, 1.34)	13,943/406,334	1.18(1.15, 1.21)

[a]Women with complete data for all covariates. [b]Adjusted for maternal age, race, education, marital status, season of conception, year of conception, prenatal care began, and median household income. [c]Adjusted for maternal age, race, education, marital status, season of conception, year of conception, prenatal care began, urbanization, and median household income.

Table S6. ORs for risk of gestational diabetes mellitus by pregnancy period of exposure from co-pollutant ($PM_{2.5}$ and O_3) models among women who gave birth from 2004 to 2005 in Florida, USA.

Exposure	Unadjusted OR (95% CI)	Adjusted OR[a] (95% CI)
Trimester 1		
$PM_{2.5}$(per 5 μg/m^3)	1.07(1.03, 1.12)	1.11(1.06, 1.16)
O_3(per 5 ppb)	1.06(1.05, 1.08)	1.08(1.05, 1.10)
Trimester 2		
$PM_{2.5}$(per 5 μg/m^3)	0.99(0.94, 1.03)	1.02(0.98, 1.07)
O_3(per 5 ppb)	1.09(1.08, 1.11)	1.12(1.10, 1.13)
Full pregnancy		
$PM_{2.5}$(per 5 μg/m^3)	1.11(1.05, 1.17)	1.10(1.04, 1.16)
O_3(per 5 ppb)	1.14(1.12, 1.17)	1.17(1.14, 1.20)

[a]Adjusted for maternal age, race, education, marital status, season of conception, year of conception, prenatal care began, urbanization, and median household income.
